# Retinitis Pigmentosa Associated With EYS Gene Mutations in Puerto Rico: A Case Series

**DOI:** 10.7759/cureus.72440

**Published:** 2024-10-26

**Authors:** Armando J Ruiz-Justiz, Leonardo J Molina Thurin, Andres Emanuelli, Natalio Izquierdo

**Affiliations:** 1 Department of Ophthalmology, University of Puerto Rico, Medical Sciences Campus, San Juan, PRI; 2 Department of Medicine, San Juan Bautista School of Medicine, Caguas, PRI; 3 Department of Surgery, University of Puerto Rico, Medical Sciences Campus, San Juan, PRI

**Keywords:** aberrant splicing, autosomal recessive retinitis pigmentosa, eyes gene, genetic analysis, rare genetic diseases, truncating mutations

## Abstract

Background: Mutations in the *EYS* (eyes shut homolog) gene are a known cause of autosomal recessive retinitis pigmentosa (arRP). Pathogenic variants in *EYS* have been associated with a more severe clinical course compared to mutations in other retinitis pigmentosa (RP)-related genes. The prevalence of *EYS*-related arRP varies among different populations. To date, no studies have described the presence of *EYS* mutations in Puerto Rican patients. This case series aims to report and characterize *EYS* mutations in RP patients from Puerto Rico.

Methods: This retrospective case series was conducted at two major ophthalmology clinics in Puerto Rico from 2019 to 2023. A chart review was performed to identify RP patients who had mutations in the *EYS* gene, identified through the Invitae Inherited Retinal Disease Panel, which evaluates more than 300 genes. Collected data included demographic information (age and gender), ocular and medical history, clinical presentation of RP, best corrected visual acuity (BCVA), and genetic testing results.

Results: Seven Puerto Rican patients, three females (43%) and four males (57%), with a clinical diagnosis of RP, were found to have pathogenic *EYS* variants. Among them, four patients (57%) carried the c.5928-2A>G variant, two (29%) had c.6794del, one (14%) had c.1211dup, and one (14%) had c.3443+1G>T. Compound heterozygosity in the *EYS* gene was observed in two patients. Additionally, three variants of unknown significance (VUS) were identified. Patients exhibited a wide range of visual acuity; however, those older than 40 were found to be legally blind.

Conclusions: This study provides evidence of *EYS*-related RP in Puerto Rican patients. Four truncating mutations in the *EYS* gene were identified, with c.5928-2A>G being the most frequent. Additionally, the novel *EYS* variant c.9263G>A (p.Gly3088Glu), classified as VUS, was identified in one patient.

## Introduction

Retinitis pigmentosa (RP) is the most common form of inherited retinal degeneration (IRD). Its estimated prevalence is one per 3,000-8,000 people worldwide [1]. Its clinical symptoms begin as nyctalopia and visual field constriction. The natural history of the disease leads to progressive degeneration of photoreceptors, severe visual impairment, and blindness. Despite being a rare disease, RP remains the primary cause of hereditary blindness in adults [1]. No risk factors for RP have been identified apart from genetic predisposition.

No significant differences in the clinical presentation or disease severity of RP have been reported across different ethnic groups. However, underserved populations may face greater barriers, including delayed diagnosis and reduced access to genetic testing [2]. These challenges often result in patients being undiagnosed or diagnosed at more advanced stages of RP, when vision impairment is already severe.

Genetic heterogeneity makes diagnosis and treatment of RP challenging. Numerous genetic mutations have been identified as causes of RP, with the distribution of these mutations varying across geographic regions [1]. Most research on RP has been conducted in Europe and among non-Hispanic white populations, leading to limited knowledge about the mutation spectrum in minority groups, such as Hispanic/Latino populations [3]. The Hispanic/Latino population in the United States has grown significantly in the past decade, comprising 19.5% of the U.S. population in 2023 [4]. However, only isolated cases of RP in Hispanic populations have been reported. 

In terms of classification, this group of diseases may be divided into syndromic or non-syndromic [1]. Mutations in more than 80 genes have been described in non-syndromic RP [1]. The eyes shut homolog (*EYS*) gene was first reported by Abd El-Aziz and coworkers in 2008 as having a role in RP [5]. This gene is considered the largest identified human eye-specific gene [5,6]. It is located in the retinitis pigmentosa-25 (RP25) locus on chromosome 6 (6q12.1-6q15) and has a length of 2 Mb [7]. The *EYS* gene is one of the principal genes associated with autosomal recessive retinitis pigmentosa (arRP). Several variants in the *EYS* gene have been found as associated with RP in studies from Europe, Asia, and Latin America [8-10]. 

Previous studies have shown that the *EYS* gene is highly expressed in the retina [5,6]. This was found through polymerase chain reaction (PCR) analysis of coding DNA (cDNA) from a variety of normal tissues and cell lines [5,6]. By using the Basic Local Alignment Search Tool (BLAST), it was found that this gene is the true ortholog of the Drosophila "eyes shut" (*EYS*) gene [6]. The *EYS* gene of Drosophila melanogaster encodes for the Spam protein, which is expressed in the open rhabdomere eye of several insect species [6]. This protein is essential for photoreceptor development and morphology in the insect eye [6].

In humans, it has been described that the *EYS* gene has a different structure from that of the Drosophila melanogaster gene [11]. The function of the *EYS* protein in the human retina is not completely understood. Several *EYS* isoforms with variable lengths and domain structures have been identified, suggesting a possible variability of functions among the different protein products from the *EYS* gene [11]. One of the possible locations of the *EYS* protein is the ciliary pocket of the photoreceptors [7]. This structure is formed by a plasma membrane invagination that separates the photoreceptor's outer and inner segments [7]. The *EYS* protein was found to stabilize ciliary axonemes in rods and cones contributing to the maintenance of photoreceptors [11]. These findings suggest that this gene is essential for the structural maintenance of the ciliary pocket and adequate photoreception function [7].

Mutations in the *EYS* gene represent 5% of arRP in the Dutch and Canadian population, 5% to 16% in Europe, and 18% to 23.5% in Japan [7]. A study of 94 unrelated Spanish families reported an estimated prevalence of 15.9% of *EYS* gene mutations in arRP patients, demonstrating the significant importance of this gene in the pathogenesis of arRP in Spain [8]. Pathogenic *EYS* variants have been associated with a more severe clinical course compared to mutations in other RP-related genes [12].

Several *EYS *variants leading to arRP have been identified ranging from insertions, deletions, nonsense or missense substitutions, and splice site mutations [7]. Although genetic predisposition is the main factor implicated in RP development, it may be potentiated by other factors such as human migration and inbreeding in geographically isolated populations.

The genetic makeup of the global ancestry of Puerto Ricans has been shaped by indigenous, African, and Spanish populations due to the Spanish colonization of the island. This may explain the presence of specific RP-related gene mutations in the Puerto Rican population originating from these groups. Among these, the *EYS* gene has been more extensively studied in the Spanish population. To our knowledge, although *EYS* mutations have been reported in Spain, no RP patients with *EYS* mutations have been documented in Puerto Rico. This study aims to report and characterize *EYS* mutations in Puerto Rican patients, contributing to improved diagnosis and earlier detection in this population.

## Materials and methods

This retrospective case series was conducted from 2019 to 2023 at two major ophthalmology clinics in Puerto Rico: the San Francisco Ophthalmology Group in San Juan and the Retina Care Clinic in Arecibo. We reviewed the medical records of patients diagnosed with RP at these clinics. All RP patients underwent routine genetic screening at diagnosis using the Invitae Inherited Retinal Disorders Panel (Invitae Corporation, San Francisco, California), which analyzes clinically relevant regions of over 300 genes associated with retinal conditions. The Invitae report classifies variants as benign, pathogenic, or of unknown significance (VUS) [13]. Variant classification is based on population data, functional studies, computational predictions, clinical data, family segregation studies, evolutionary conservation, and evidence from databases such as ClinVar and in silico tools [13].

The inclusion criteria for this study were a confirmed diagnosis of RP and the presence of pathogenic mutations in the *EYS* gene. Exclusion criteria included incomplete medical records, absence of genetic testing results, pathogenic mutations in genes other than *EYS*, or the presence of other ocular or systemic conditions that could interfere with the diagnosis. All patient information was anonymized to ensure confidentiality.

Seven patients with RP who had pathogenic mutations in the *EYS* gene were included in the study. Information collected from medical records included demographic data (age and gender), past ocular and medical history, the clinical presentation of RP, best-corrected visual acuity (BCVA), and genetic testing results. A descriptive analysis was conducted to calculate the frequency of *EYS* variants identified in our cohort. This analysis aimed to quantify the occurrence of specific *EYS* mutations within the studied population. The ClinVar database was used to corroborate each variant classification. The combined annotation-dependent depletion (CADD) score of each variant was obtained from the UCSC Genome Browser (https://genome.ucsc.edu) to assess the potential pathogenicity of each *EYS* variant present in our cohort. 

In accordance with the ethical principles governing medical research, the Institutional Review Board of the University of Puerto Rico Medical Sciences Campus conducted a thorough review of the research protocol. The protocol received approval (2404222793), confirming adherence to ethical standards and patient confidentiality in this retrospective study.

## Results

Patient 1

A 34-year-old male patient with a past medical history of RP since age 21, diabetes mellitus (DM), diabetic retinopathy, bilateral vitreous degeneration, bilateral dry eye syndrome, and hypothyroidism. He was referred to our eye clinic for evaluation of RP progression and genetic testing. The patient underwent a comprehensive ophthalmic evaluation by at least one of the authors (NJI).

The BCVA was 20/60 in both eyes. Intraocular pressure (IOP) was 17 mmHg in both eyes. Upon fundus examination, the patient had pale optic discs, intact macula, arteriolar attenuation, and peripheral bony spicules in both eyes.

Macular optical coherence tomography (OCT) showed a macular thickness of 248 microns and 249 microns in the right and left eye, respectively. Total macular volume was 7.6 mm³ and 7.2 mm³ in the right and left eye, respectively. Upon visual field testing, the mean deviation (MD) was -32.01 dB (p<0.5%) in the right eye and -33.23 dB (p<0.5%) in the left eye; and the pattern standard deviation (PSD) was 3.59 dB (p<0.5%) and 1.76 dB (p<0.5%) in the right and left eye, respectively. 

Full-field electroretinogram (ERG) was done using corneal contact lens electrodes. It recorded hardly discernible scotopic and photopic ERG responses. Flicker responses and oscillatory responses were diminished in amplitude. These findings indicate involvement of the retinal pigment epithelium (RPE) and photoreceptors, consistent with progressive rod-cone dystrophy.

A patient’s saliva sample underwent gene sequencing and deletion/duplication analysis using next-generation sequencing (NGS) (Invitae Corporation, San Francisco, California). The homozygous pathogenic variant c.5928-2A>G (splice acceptor) in the *EYS* gene (rs181169439) was identified, as shown in Table 1.

**Table 1:** Summary of patients' demographic data, BCVA at diagnosis, EYS variants, and their classification. M: male, F: female, BCVA: best corrected visual acuity, OU: both eyes, OD: right eye, OS: left eye, CF: counting fingers, HM: hand motion, LP: light perception, VUS: variants of unknown significance

Patient ID	Sex	Age	BCVA at Diagnosis	EYS Variant	Variant Classification	Zygosity
1	M	34	20/60 OU	c.5928-2A>G (splice acceptor)	Pathogenic	Homozygous
2	M	35	20/40 OD, 20/30 OS	c.5928-2A>G (Splice acceptor)	Pathogenic	Homozygous
3	F	26	20/25 OU	c.3443+1G>T (Splice donor)	Pathogenic	Heterozygous
				c.1211dup (p.Asn404Lysfs*3)	Pathogenic	Heterozygous
				c.3250A>C (p.Thr1084Pro)	VUS	Heterozygous
				c.4402G>C (p.Asp1468His)	VUS	Heterozygous
4	M	45	LP OD, HM OS	c.6794del (p.Pro2265Glnfs*46)	Pathogenic	Homozygous
5	F	41	LP OD, 20/200 OS	c.5928-2A>G (Splice acceptor)	Pathogenic	Homozygous
6	F	83	CF OD, 20/400 OS	c.6794del (p.Pro2265Glnfs*46)	Pathogenic	Homozygous
7	M	46	HM OD, 20/200 OS	c.5928-2A>G (Splice acceptor)	Pathogenic	Heterozygous
				c.9263G>A (p.Gly3088Glu)	VUS	Heterozygous

Patient 2

A 35-year-old male patient with a past ocular history of RP and bilateral posterior subcapsular cataract. The patient underwent a comprehensive ophthalmic evaluation by at least one of the authors (NJI).

The BCVA was 20/40 and 20/30 in the right and left eye, respectively. IOP was 14 mmHg in both eyes. Upon fundus examination, the patient had pale optic discs, intact macula, arteriolar attenuation, and peripheral bony spicules in both eyes.

Visual field testing showed MD of -25.18 dB (p<0.5%) in the right eye and -23.95 dB (p<0.5%) in the left eye, and a PSD of 9.07 dB (p<0.5%) and 9.45 dB (p<0.5%) in the right and left eye, respectively.

A patient’s saliva sample underwent gene sequencing and deletion/duplication analysis using NGS (Invitae Corporation, San Francisco, California). The homozygous pathogenic variant c.5928-2A>G (splice acceptor) in the *EYS* gene (rs181169439) was identified, as shown in Table 1. 

Patient 3

A 26-year-old female patient with an ocular history of RP and bilateral posterior subcapsular cataract was referred to our eye clinic for evaluation of RP progression, genetic testing, and cataract management. The patient underwent a comprehensive ophthalmic evaluation by at least one of the authors (NJI).

The BCVA was 20/25 in both eyes. IOP was 16 mmHg in both eyes. Upon fundus examination, the patient had pale optic discs, intact macula, arteriolar attenuation, and peripheral bony spicules in both eyes.

Upon visual field testing, MD was -22.73 dB (p<0.5%) and -24.06 dB (p<0.5%) in the right and left eye, respectively; and PSD was 13.54 dB (p<0.5%) in the right eye and 12.85 dB (p<0.5%) in the left eye.

Full-field ERG showed absent scotopic and photopic ERG responses. Flicker and oscillatory responses were diminished in amplitude. These findings indicate the involvement of the RPE and photoreceptors, consistent with progressive rod-cone dystrophy.

A patient’s saliva sample underwent gene sequencing and deletion/duplication analysis using NGS (Invitae Corporation, San Francisco, California). Four variants were identified in the *EYS* gene: the heterozygous pathogenic variant c.1211dup (p.Asn404Lysfs*3) (rs764163418), the heterozygous pathogenic variant c.3443+1G>T (splice donor) (rs373441420), and two heterozygous variants of uncertain significance (VUS), c.3250A>C (p.Thr1084Pro) (rs778646190) and c.4402G>C (p.Asp1468His) (rs778752557), as shown in Table 1. 

Patient 4

A 45-year-old male patient with a medical history of DM and hypercholesterolemia, and an ocular history of glaucomatous optic atrophy in both eyes, bilateral vitreous degeneration, and RP. He was referred to our eye clinic for evaluation of RP progression and genetic testing. The patient underwent a comprehensive ophthalmic evaluation by at least one of the authors (NJI).

The BCVA was light perception (LP) and hand motion (HM) in the right and left eye, respectively. IOP was 13 mmHg in both eyes. Upon fundus examination, the patient had pale optic discs, intact macula, arteriolar attenuation, and peripheral bony spicules in both eyes.

The macular OCT showed a macular thickness of 121 microns and 108 microns in the right and left eye, respectively. Total macular volume was 7.3 mm³ and 7.2 mm³ in the right and left eye, respectively.

Upon visual field testing, MD was -32.99 dB (p<0.5%) in the right eye, and PSD was 1.88 dB (p<0.5%) in the right eye. Visual field testing in the left eye was impossible due to poor vision.

A patient’s saliva sample underwent gene sequencing and deletion/duplication analysis using NGS (Invitae Corporation, San Francisco, California). The homozygous pathogenic variant c.6794del (p.Pro2265Glnfs*46) in the *EYS* gene (rs758109813) was identified, as shown in Table 1. 

Patient 5

A 41-year-old female patient with an ocular history of nuclear sclerotic cataract (NSC) in both eyes and RP. She was referred to our eye clinic for evaluation of RP progression and genetic testing. The patient underwent a comprehensive ophthalmic evaluation by at least one of the authors (AE).

The BCVA was LP and 20/200 in the right and left eye, respectively. IOP was 13 mmHg and 20 mmHg in the right and left eye, respectively. Upon fundus examination, the patient had pale and atrophic optic discs, arteriolar attenuation, and peripheral bony spicules in both eyes.

A patient’s saliva sample underwent gene sequencing and deletion/duplication analysis using NGS (Invitae Corporation, San Francisco, California). The homozygous pathogenic variant c.5928-2A>G (Splice acceptor) in the *EYS* gene (rs181169439) was identified, as shown in Table 1. 

Patient 6

An 83-year-old female patient with a medical history of DM type 2, hypercholesterolemia, and hypertension, and an ocular history of RP was diagnosed two years ago. She had undergone cataract surgery. The patient was referred to our eye clinic for evaluation of RP progression and genetic testing. The patient underwent a comprehensive ophthalmic evaluation by at least one of the authors (AE).

The BCVA was counting fingers (CF) and 20/400 in the right and left eye, respectively. IOP was 11 mmHg and 12 mmHg in the right and left eye, respectively. Upon fundus examination, the patient had pale and atrophic optic discs, arteriolar attenuation, and peripheral bony spicules in both eyes.

Upon visual field testing, MD was -16.88 dB (p<0.5%) and -18.00 dB (p<0.5%) in the right and left eye, respectively.

A patient’s saliva sample underwent gene sequencing and deletion/duplication analysis using NGS (Invitae Corporation, San Francisco, California). The homozygous pathogenic variant c.6794del (p.Pro2265Glnfs*46) was identified in the *EYS* gene (rs758109813), as shown in Table 1. 

Patient 7

A 46-year-old male patient with an ocular history of RP was referred to our eye clinic for evaluation of RP progression and genetic testing. The patient underwent a comprehensive ophthalmic evaluation by at least one of the authors (AE).

The BCVA was HM and 20/200 in the right and left eye, respectively. IOP was 10 mmHg and 19 mmHg in the right and left eye, respectively. Upon fundus examination, the patient had pale and atrophic optic discs, arteriolar attenuation, and peripheral bony spicules in both eyes.

A patient’s saliva sample underwent gene sequencing and deletion/duplication analysis using NGS (Invitae Corporation, San Francisco, California). The heterozygous pathogenic variant c.5928-2A>G (splice acceptor) (rs181169439) and the heterozygous VUS c.9263G>A (p.Gly3088Glu) (rs913465684), were identified in the *EYS* gene as shown in Table 1.

This case series shows the presence of *EYS*-related arRP in Puerto Rico. Patients' demographic and clinical characteristics have been summarized in Table 1.

The patients ranged in age from 26 to 83 years at the time of diagnosis. BCVA ranged from 20/25 in both eyes (patient 3) to LP (patients 4 and 5). The patients’ ages at the time of diagnosis played a role in this difference. Most patients were referred to our clinics for RP evaluation when the disease was already advanced, making it challenging to determine the age of disease onset. Out of seven patients, three were female (43%) and four were male (57%). All patients were Puerto Ricans. Upon reviewing their medical histories, three out of seven (43%) were found to have DM. Other medical conditions such as hypertension, hypothyroidism, and hypercholesterolemia were observed in some patients.

Among the seven patients with pathogenic *EYS *mutations, four (57%) carried the variant c.5928-2A>G, two (29%) had c.6794del, one (14%) had c.1211dup, and one (14%) had c.3443+1G>T, as shown in Table 2. The most common pathogenic *EYS* variant in our cohort was c.5928-2A>G. Of the four patients with the c.5928-2A>G variant, three were found to be homozygous. Additionally, three VUS were identified: c.3250A>C (p.Thr1084Pro), c.4402G>C (p.Asp1468His), and c.9263G>A (p.Gly3088Glu). 

**Table 2:** Frequency of EYS variants found in our cohort, their classification, and CADD score. rsID: variant identification number, VUS: variants of unknown significance, CADD: combined annotation-dependent depletion

*EYS* Variant	Variant rsID	Frequency	Variant Classification	CADD Score
c.5928-2A>G	rs181169439	4/7 (57%)	Pathogenic	29.8
c.6794del	rs758109813	2/7 (29%)	Pathogenic	20.9
c.3443+1G>T	rs373441420	1/7 (14%)	Pathogenic	34
c.1211dup	rs764163418	1/7 (14%)	Pathogenic	18.57
c.9263G>A	rs913465684	1/7 (14%)	VUS	23.6
c.4402G>C	rs778752557	1/7 (14%)	VUS	21.3
c.3250A>C	rs778646190	1/7 (14%)	VUS	6.46

The CADD score is a predictive metric for assessing the deleteriousness of genomic variations, including insertions, deletions, and single nucleotide variants [14]. By integrating multiple annotations and comparing naturally occurring variants with hypothetical mutations, the CADD score has shown a strong association with allelic diversity and pathogenicity [14]. Variants with CADD scores above 20 are considered among the top 1% of potentially harmful mutations in the human genome [14]. The UCSC Genome Browser (https://genome.ucsc.edu) estimates CADD scores (version 1.7) for the *EYS* variants identified in our cohort as follows: c.5928-2A>G with a score of 29.8, c.6794del with 20.9, c.3443+1G>T with 34, c.1211dup with 18.57, c.9263G>A with 23.6, c.4402G>C with 21.3, and c.3250A>C with 6.46, as shown in Table 2 [15]. 

## Discussion

The *EYS* gene has been associated with autosomal recessive retinitis pigmentosa (arRP). Several truncating mutations in the *EYS* gene leading to aberrant splicing, early stop codon generation, or changes in protein structure have been documented [16]. In this case series, seven Puerto Rican patients with a clinical diagnosis of RP and pathogenic *EYS* gene mutations as the most likely etiology were identified and studied. To our knowledge, this is the first study reporting the presence of *EYS*-related RP in Puerto Rico. 

The pathogenic *EYS* variant c.5928-2A>G was first reported to play a role in RP by Gonzalez del Pozo and co-workers in a cohort of Spanish patients [17]. By using the Berkeley Drosophila Genome Project (BDGP) website, they determined that this variant causes a splicing site mutation. McGuigan et al. (2017) reported a case of a compound heterozygous male Hispanic patient with the pathogenic c.5928-2A>G and c.4829_4832delCATT variants in the *EYS* gene [18]. The patient was initially evaluated at age 19 with a visual acuity of 20/20 and mid-peripheral scotomas in both eyes. By age 37, his visual acuity had deteriorated to only residual central and peripheral islands, progressing to just a central island by age 42, and no measurable peripheral cone function by age 46, suggesting a rapid RP progression associated with the c.5928-2A>G variant. The clinical progression of RP in this patient aligns with the visual outcomes of the three patients in our cohort who were homozygous for the pathogenic c.5928-2A>G variant (patients 1, 2, and 5). The visual acuity of those patients varied from 20/30 to light perception, showing a diverse range of severity in these patients possibly related to age. Patient 1 and patient 2 who were 34 and 35 years old, respectively, had a decreased visual acuity ranging from 20/30 to 20/60. However, patient 5 had a visual acuity of light perception in the right eye and 20/200 in the left eye at age 41, indicating a severe form of RP progression by the 4th decade. The visual deterioration in these cases is similar to those reported by McGuigan et al. (2017). Patient 1, patient 2, and patient 5 suggest a severe and rapidly progressive form of arRP associated with the c.5928-2A>G variant. This variant has a CADD score of 29.8 (version 1.7), suggesting a deleterious effect of this mutation [15]. Since this variant was first reported in the Spanish population, its presence in arRP patients in Puerto Rico could be explained by the Spanish population's influence on the genetic makeup of Puerto Ricans due to the Spanish colonization of the island over four centuries [5]. 

RP has been reported in compound heterozygotes [18]. Patient 7, in addition to being heterozygous for the c.5928-2A>G variant, was also found to carry the heterozygous VUS c.9263G>A (p.Gly3088Glu) in the *EYS* gene, suggesting possible compound heterozygosity as the etiology of RP in this patient. The c.9263G>A variant replaces a highly conserved glycine residue with glutamic acid at codon 3088 of the *EYS* protein. This variant has not been previously reported in individuals with *EYS*-related conditions, making this the first known case of an RP patient carrying the c.9263G>A (p.Gly3088Glu) variant. Predictive algorithms performed by Invitae Corporation were either unavailable or yielded conflicting results regarding the impact of this missense change, leading to its classification as a VUS. However, the CADD score of this variant is 23.6 (version 1.7), suggesting a possible deleterious effect [15]. This finding strengthens the hypothesis that this novel variant may contribute to RP. At the age of 46, patient 7 was legally blind, with a visual acuity of hand motion in the right eye, and 20/200 in the left eye. The severe visual impairment in this patient may have resulted from the combined effects of the *EYS* variants c.5928-2A>G and c.9263G>A. Further research is required to determine the association between the c.9263G>A variant and RP. 

Audo et al. (2010) first reported the c.6794del (p.Pro2265Glnfs*46) variant in *EYS *as being associated with arRP [19]. Through direct sequencing of the *EYS* gene in 186 French RP patients, they identified several novel mutations, including c.6794del (p.Pro2265Glnfs*46). In their study, only one patient carried this variant. Interestingly, because the patient was adopted and their ethnicity was not disclosed, it cannot be concluded that this *EYS* variant originated in the French population. The c.6794del (p.Pro2265Glnfs*46) variant results in a sequence change in exon 34 of the *EYS* gene, leading to a premature translational stop signal and the generation of an absent or disrupted protein product. Previous studies have demonstrated that loss of function in the *EYS* gene is pathogenic and associated with arRP [2]. Additionally, the CADD score of this variant is 20.9 (version 1.7), supporting its classification as pathogenic [15]. In our cohort, patient 4 and patient 6 were both homozygous for the pathogenic c.6794del (p.Pro2265Glnfs*46) variant. At age 45, patient 4 had a visual acuity of light perception in the right eye and hand motion in the left. At age 83, patient 6 had a visual acuity of CF in the right eye and 20/400 in the left eye. These cases suggest that the c.6794del (p.Pro2265Glnfs*46) variant may be associated with a severe form of arRP. Our findings are consistent with the literature, further reinforcing the role of the c.6794del (p.Pro2265Glnfs*46) variant in arRP. 

Variants leading to splicing site mutations in *EYS* have been documented in arRP [20]. The c.3443+1G>T (splice donor) variant affects a donor splice site in intron 22 of the *EYS* gene. This sequence change is expected to disrupt RNA splicing, resulting in an absent or disrupted protein product [20]. Based on algorithms used at Invitae aimed at predicting the effect of genomic sequence changes on RNA splicing, this variant may compromise the consensus splice site. Disruption of this splice site has been observed in patients with retinal disease [20]. This is consistent with previous studies demonstrating that donor and acceptor splice site variants typically lead to a loss of protein function [7,16]. The CADD score of this variant is 34 (version 1.7), supporting its classification as pathogenic [15]. The pathogenic variant c.1211dup (p.Asn404Lysfs*3) at exon 8 creates a premature translational stop signal in the *EYS* gene, which results in an absent or disrupted protein product. This variant has also been observed in patients with arRP [21]. While the CADD score (version 1.7) of this variant is lower than 20 (18.57), algorithmic models at Invitae support its pathogenicity. In our cohort, patient 3 was heterozygous for the pathogenic variants c.3443+1G>T (splice donor) and c.1211dup (p.Asn404Lysfs*3). This represents a case of compound heterozygosity, where different pathogenic variants in the *EYS* gene may contribute to the development of RP. Additionally, the VUS c.4402G>C identified in this patient may contribute to further destabilization of the final *EYS* protein product, given its CADD score of 21.3, suggesting a potential deleterious effect [15]. In contrast, the VUS c.3250A>C, also found in this patient, has a CADD score of 6.46, making it less likely to be pathogenic [15]. Further research is required to clarify the roles of these VUS in RP.

DM and RP may occur independently. However, the co-occurrence of both conditions is observed in four syndromes: Bardet-Biedl, Alström, Kearns-Sayre, and Wolfram syndromes [22]. Other co-morbidities in patients with these syndromes include obesity, deafness, hypogonadism, polydactyly, renal abnormalities, and/or intellectual disabilities [22]. In this study, three out of seven patients (43%) had both DM and RP, but none displayed phenotypic characteristics or findings consistent with syndromic RP. Given the high prevalence of DM in Puerto Rico (20.1%), which exceeds the global prevalence (10.5%), it is likely that the diagnosis of DM and RP in these patients is unrelated [23]. Currently, no direct association or shared heritability has been identified between DM and RP. However, further research exploring the potential influence of DM on the severity and progression of RP would be beneficial in understanding the implications of this coexistence and in providing more comprehensive care for these patients.

Visual symptoms in RP typically manifest during the second or third decade of life [1,7]. Mutations in the *EYS* gene have been associated with poorer visual outcomes compared to mutations in other RP-related genes [12]. Our findings support this observation, as all patients in our cohort over the age of 40 were legally blind, further highlighting the potential role of *EYS* mutations in severe and rapidly progressing forms of RP. In terms of sex distribution, no significant differences in the prevalence of *EYS*-related RP between males and females have been reported [7]. Consistent with this, our study found a sex distribution of 43% female (three out of seven) and 57% male (four out of seven). 

The primary limitation of this study is the small sample size, which is primarily attributed to the rarity of the disease. This may limit the generalizability of our findings. Additionally, the retrospective design may introduce selection biases, as patients with more severe symptoms are more likely to undergo genetic testing. Future studies with larger sample sizes, including patients from additional eye clinics in Puerto Rico and the U.S., are needed to validate our findings and broaden the understanding of *EYS*-associated RP. A meta-analysis of similar studies could also provide more comprehensive insights into the prevalence and clinical impact of *EYS* variants across different populations.

## Conclusions

Our case series provides evidence of *EYS*-associated RP in Puerto Rican patients, highlighting its clinical heterogeneity. Patients exhibited a wide range of visual acuity; however, those older than 40 were found to be legally blind, suggesting a potential role for *EYS* mutations in more severe forms of RP. Several truncating mutations in the *EYS* gene were identified, with the c.5928-2A>G variant, originally reported in the Spanish population, being the most common in our cohort. Additionally, we identified the novel *EYS* variant c.9263G>A (p.Gly3088Glu) in one of our patients, which has been classified as VUS. However, this variant has a CADD score of 23.6, suggesting a possible deleterious effect. Further research and molecular studies are required to clarify the role of this variant in RP. 
